# Correlation between gastroesophageal flap valve abnormality and novel parameters in patients with gastroesophageal reflux disease symptoms by the lyon consensus

**DOI:** 10.1038/s41598-021-94149-w

**Published:** 2021-07-23

**Authors:** Zihao Guo, Yanhong Wu, Yutao Zhan, Chuan Zhang

**Affiliations:** Department of Gastroenterology, Beijing Tong Ren Hospital, Capital Medical University, No.1, Dongjiaominxiang, Dongcheng District, Beijing, 100730 China

**Keywords:** Gastrointestinal diseases, Gastrointestinal system

## Abstract

Gastroesophageal flap valve (GEFV) grading is a simple and reproducible parameter. There is limited information about the association between GEFV abnormality and novel parameters in patients with gastroesophageal reflux disease(GERD) symptoms by the Lyon Consensus. To investigate the value of GEFV grading in GERD, the clinical data of 320 patients with GERD symptoms who underwent endoscopy, 24-h multichannel intraluminal impedance-pH (MII-pH) monitoring, and high-resolution manometry (HRM) were retrospectively analyzed. The percentage of acid exposure time (AET%)(4.2 [1.5–7.4] vs. 1.3 [0.3–4.2], *P* < 0.001) and the proportion of abnormal esophagogastric junction (EGJ) morphology (71 [87.7%] vs. 172 [72.0%], *P* = 0.011) were significantly higher, while the mean nocturnal baseline impedance (MNBI) (2068.3 [1658.4–2432.4] vs. 2228.5 [1794.8–2705.3]Ω, *P* = 0.012) and post-reflux swallow-induced peristaltic wave index (PSPWI) (19.7 [13.9–29.0] vs. 33.3 [25.0–44.0]%, *P* < 0.001) were significantly lower in the abnormal GEFV group compared with the normal GEFV group. AET% and EGJ morphology showed positive correlations with GEFV grade, while PSPWI and MNBI showed negative correlations. Patients with an abnormal GEFV had a significantly greater risk of conclusive evidence of GERD compared to those with a normal GEFV (OR 3.035, 95% CI 1.758–5.240, *P* < 0.001). Further, when identifying patients with conclusive evidence of GERD, abnormal GEFV had a specificity of 80.4% (95% CI 75.3–85.5%). GEFV grading might be regarded as supportive evidence for GERD diagnosis.

## Introduction

Gastroesophageal reflux disease (GERD) is a common clinical condition. Although it is a benign disease, longstanding GERD can lead to Barrett's esophagus (BE), a premalignant condition^1^. Hence, accurate and timely diagnosis of GERD is important. The Lyon Consensus was developed to aid the diagnosis of GERD based on 24-h multichannel intraluminal impedance-pH (MII-pH), upper gastrointestinal endoscopy, and high-resolution manometry (HRM). Accordingly, the evidence for GERD is classified as follows: conclusive evidence for pathologic reflux, borderline or inconclusive evidence, adjunctive or supportive evidence, and evidence against pathological reflux^2^.

MII-pH monitoring is used increasingly often for diagnosing GERD. The mean nocturnal baseline impedance (MNBI) and post-reflux swallow-induced peristaltic wave index (PSPWI) are two novel parameters detected on MII-pH and regarded as supportive parameters for detecting GERD^2^. The MNBI reflects the reflux-induced impairment of mucosal integrity^3,4^. On the other hand, the PSPWI highlights the integrity of primary peristalsis initiated by reflux^4^.

The esophagogastric junction (EGJ) is important for the prevention of GERD^5^. The gastroesophageal flap valve (GEFV) serves as the anti-reflux barrier and is easily visualized with a retroflexed endoscope with an excellent inter-observer agreement^6^. Previous studies have found that GEFV grade is positively associated with acid reflux and could reflect EGJ morphology^7^.

However, the GEFV is not included in the Lyon Consensus, and studies on the potential associations between GEFV grading and GERD classification by the Lyon Consensus, mucosal integrity, esophageal peristaltic function, and EGJ are limited. In the present study, we aimed to assess the correlations between GEFV grading and the classification of GERD by the Lyon Consensus and to explore the relationship of GEFV grading with the MNBI and PSPWI, EGJ morphology, EGJ tone, and esophageal body motility.

## Methods

### Patient selection

A retrospective chart review of adult patients with GERD symptoms (heartburn and/or regurgitation) who underwent endoscopy, HRM, and 24-h MII-pH at Beijing Tong Ren Hospital from August 2015 to January 2020 was conducted. The exclusion criteria were: inadequate or incomplete studies; pregnancy; previous thoracic, esophageal or gastric surgery; the presence of infectious, eosinophilic esophagitis or scleroderma; achalasia cardia, and EGJ outflow obstruction and major esophageal motility disorders such as absent contractility. The Medical Ethics Committee of Beijing Tong Ren Hospital approved the study (Approved Document Number: trxhzcfa01). All patients had signed informed consent for the endoscopy, MII-pH, HRM, and use of data for research purposes. And all methods were carried out in accordance with relevant guidelines and regulations.

### Endoscopic examination

All patients underwent upper gastrointestinal (GI) endoscopy using a GIF-260 upper GI endoscope (Olympus, Hamburg, Germany) under intravenous anesthesia or topical anesthesia. Esophagitis was diagnosed and graded by the Los Angeles classification^8^. Barrett’s esophagus (BE) was diagnosed by confirmation of intestinal metaplasia by pathology. Each GEFV was graded using Hill's classification from grade I to IV as described previously^6^. A GEFV that remained tightly snug to the endoscope was categorized as grade I. A GEFV that opened and closed with respiration was considered grade II. A GEFV that failed to remain snug to the endoscope was rated as grade III. If a wide-open diaphragmatic hiatus was observed by endoscopy, the GEFV was categorized as grade IV^6^. All endoscopists in the department had attended a previous training workshop on GEFV evaluation and were encouraged to assess GEFV grading during endoscopic examination. All endoscopic procedures in our study were performed by one experienced endoscopist (Z.H.G.) who had experience performing upper gastrointestinal endoscopy with at least 5000 procedures over the last 5 years, and GEFV grading was determined during endoscopic examination. To ensure consistency of GEFV grading, all the clear photographs of GEFV of enrolled patients were reviewed again by the other author (Y.T.Z.), who was blind to the patient information. If disagreement occurred, a senior endoscopist (C.Z.) would participate in making the final decision.

### Esophageal HRM

All patients were instructed to stop proton pump inhibitors, prokinetic antagonists, and H2 receptors at least 2 weeks before esophageal HRM and reflux monitoring. HRM was performed after an overnight fast using a 36-channel trans-nasal solid-state catheter system with circumferential sensors 1 cm apart (Medical Measurement Systems Inc. [MMS], Williston, VT, USA). Esophageal motility was assessed by using 5 mL of ambient temperature water at 30-s intervals for at least 10 water swallows as previously described^9^. The Chicago Classification version 3.0 (CCv3.0) criteria were used to evaluate each HRM study (EGJ morphology, EGJ tone, and esophageal body contractility and motility)^10^. The EGJ morphology was determined by the relation between the lower esophageal sphincter (LES) and crural diaphragm (CD). EGJ-contractile integral (EGJ-CI), expiratory EGJ pressure (EGJP-exp), and inspiratory EGJ pressure (EGJP-insp) were calculated as previously described^10,11^. Esophageal body motility was classified as follows: normal peristalsis, ineffective esophageal motility (IEM), and fragmented peristalsis^10^.

### MII-pH recording

Immediately after HRM, the pH-impedance catheter (eight impedance rings and one pH ring, Ref. No 261A; Given Imaging, Los Angeles, CA, USA) was placed. Data from the pH-impedance monitor were downloaded and analyzed using the MMS database software. All data were initially identified by the software and subsequently verified and calculated by two of the authors (Z.H.G. and Y.H.W.) for accuracy. The total number of reflux events and the total acid exposure time (AET) were recorded. Also, symptom index (SI) and symptom association probability (SAP) were determined. SI ≥ 50% or SAP ≥ 95% was considered as positive, while SAP < 95% and SI < 50% was labeled as negative^12^. Additionally, PSPWI and MNBI were determined as described previously^13,14^.

### Group definitions

Patients were classified into three groups according to the Lyon Consensus as follows: (1) conclusive evidence for GERD (conclusive GERD): presence of erosive esophagitis (EE) LA grades C or D, long-segment BE or peptic strictures or AET > 6%; (2) inconclusive or borderline evidence for GERD (inconclusive or borderline GERD): LA grades A or B esophagitis, AET between 4 and 6%, or reflux episodes 40–80. In this group, adjunctive evidence included low MNBI, low PSPWI, reflux episodes > 80, and reflux-symptom association. (3) evidence against pathologic reflux (against GERD): normal endoscopic findings as well as AET < 4% and reflux episodes < 40 on pH-impedance monitoring when patients are off PPIs^2^. Among these patients, those with normal endoscopic findings, normal AET, negative SAP, and SI in the setting of esophageal symptoms were labeled to have FH, and those with normal endoscopic findings, normal AET, but positive SAP or SI were considered to have RH based on Rome IV criteria^15^.

Patients were grouped according to GEFV grading as follows: normal GEFV (grades I and II) and abnormal GEFV (grades III and IV)^6^.

### Statistical analysis

The SPSS version 23.0 (SPSS Inc., Chicago, IL, USA) and Prism software version 6.0 (GraphPad Software Inc., San Diego, CA, USA) were used for analysis. Continuous data were expressed as mean ± standard deviation (SD) and compared using either one-way analysis of variance (ANOVA) among the four groups or t-test between two groups if the data were normally distributed. Continuous data with a skewed distribution were reported as median (interquartile range [IQR]) and compared using either Kruskal–Wallis H test among four groups or Mann–Whitney U test between two groups. Qualitative data were reported as numbers (percentages), and the chi-squared test was used to compare them. Spearman's correlation analysis was used to determine the association between MII-pH parameters and GEFV grade, and the association between HRM parameters and GEFV grade. Inter-observer agreement of GEFV grading was evaluated using weighted kappa statistics with linear weights. Logistic regression analysis was used to assess whether sedation was a risk fator for abnormal GEFV, and whether abnormal GEFV was a risk factor for conclusive evidence of GERD. And diagnostic values (accuracy, sensitivity, specificity, and predictive values) of abnormal GEFV to identify patients with conclusive evidence of GERD were calculated. A value of *P* < 0.05 was considered to be statistically significant.

## Results

### Patient characteristics and distribution of GEFV grades among patients with conclusive evidence of GERD, inconclusive or borderline evidence of GERD, and evidence against GERD

**Figure 1:**
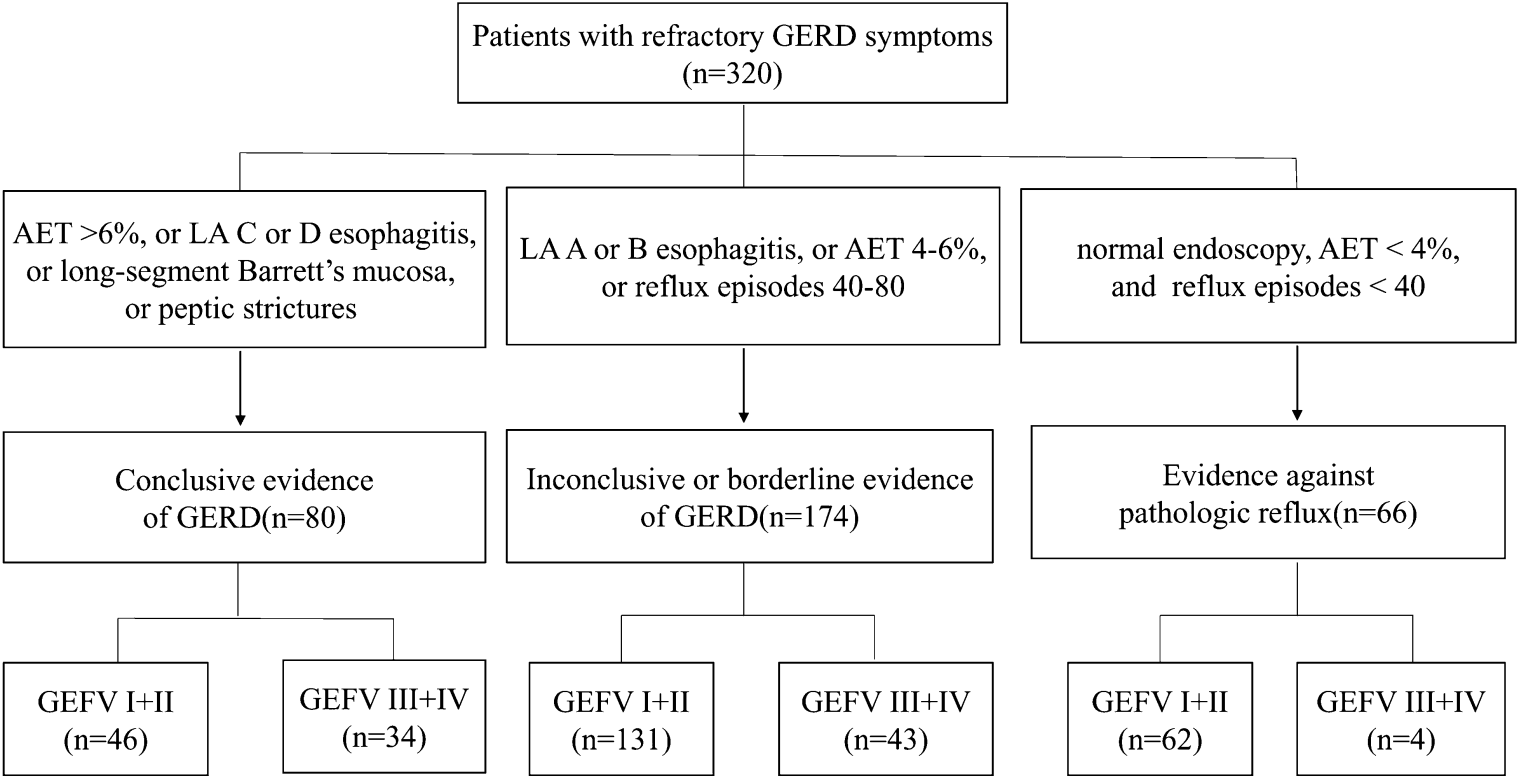
Flow chart of 320 patients with GERD symptoms as classified by the Lyon Consensus. *GERD* gastroesophageal reflux disease, *AET* acid exposure time, *LA* Los Angeles, *GEFV* gastroesophageal flap valve.

A total of 320 Chinese patients who satisfied the selection criteria were included. Of these, 80 (25.0%) patients were classified into the conclusive evidence group, 174 (54.4%) patients into the inconclusive or borderline evidence group, and 66 (20.6%) patients into the evidence against pathologic reflux group. Among patients with evidence against pathologic reflux, 19 (28.8%) patients had RH, and 47 (71.2%) patients had FH (Table 1 and Fig. 1). The proportions of male patients and mean body mass index (BMI) were significantly higher in patients with conclusive evidence of GERD than in patients with inconclusive or borderline evidence of GERD and in patients with evidence against pathological reflux (39 [48.8%] *vs.* 56 [31.5%] and *vs.* 14 [21.2%]; 24.7 ± 3.4 vs. 23.4 ± 3.7 and vs. 23.3 ± 4.0, respectively; all *P* < 0.05) (Table 1). The median ages of the three groups were similar.

**Table 1:** Demographic and clinical characteristics of 320 patients with GERD symptoms according to the lyon consensus.

	Conclusive evidence of GERD (Group 1, n = 80)	Inconclusive evidence of GERD (Group 2, n = 174)	Evidence against pathologic reflux (Group 3, n = 66)	P value	P value (Group 1 vs. Group 2)	P value (Group 1 vs. Group 3)	P value (Group 2 vs. Group 3)
Male gender, n (%)	39 (48.8%)	56 (31.5%)	14(21.2%)	0.002	< 0.05	< 0.05	> 0.05
Age (years)	61.5(50.3–69.0)	61.0(52.0–67.0)	56.0(51.0–64.0)	0.446	> 0.05	> 0.05	> 0.05
BMI, Mean ± SD	24.7 ± 3.4	23.4 ± 3.7	23.3 ± 4.0	0.020	0.008	0.028	0.958
				< 0.001	< 0.05	< 0.05	< 0.05
GEFV I (n = 94)	10(12.5%)	41(23.6%)	43(65.2%)				
GEFV II (n = 145)	36(45.0%)	90(51.7%)	19(28.8%)				
GEFV III (n = 40)	8(10.0%)	28(16.1%)	4(6.1%)				
GEFV IV (n = 41)	26(32.5%)	15(8.6%)	0				
				< 0.001	< 0.05	< 0.05	< 0.05
GEFV I + II (n = 239)	46(57.5%)	131(75.3%)	62(93.9%)				
GEFVIII + IV(n = 81)	34(42.5%)	43(24.7%)	4(6.1%)				

Values are presented as the median and interquartile range for age, means and standard deviation for BMI, and n (%) for male gender and GEFV grades.

*GERD* gastroesophageal reflux disease, *BMI* body mass index, *GEFV* gastroesophageal flap valve.

The difference was significant when *P* < 0.05.

The weighted kappa coefficient for GEFV grading was 0.921 (95% CI 0.892–0.951) between the two investigators. The upper GI endoscopy was performed in 67 patients under intravenous anesthesia and 253 patients under topical anesthesia. Among the 320 patients, 24 patients had undergone twice EGD examination under intravenous anesthesia and topical anesthesia respectively within 2 years, which were performed by the same endoscopist. Among the 24 patients, the GEFV grading of 2 patients was not consistent between intravenous anesthesia and topical anesthesia (one patient with GEFV I under intravenous anesthesia but with GEFV II under topical anesthesia; the other patient with GEFV II under intravenous anesthesia but with GEFV I under topical anesthesia). The concordance rate of GEFV grading was 91.7% between intravenous anesthesia and topical anesthesia. And sedation was not a risk factor for abnormal GEFV (OR 0.582, 95% CI 0.294–1.152, *P* = 0.120).

As shown in Table 1, abnormal GEFV (GEFV III + IV) was found in 42.5% of the patients with conclusive evidence of GERD, and among 25.3% of all study patients. The proportion of abnormal GEFV was significantly higher in the conclusive evidence of GERD group than in the inconclusive or borderline evidence of GERD group and in the evidence against GERD group (34 [42.5%] *vs.* 43 [24.7%] *vs.* 4 [6.1%]; both *P* < 0.05). In addition, patients with inconclusive or borderline evidence of GERD had a significantly higher proportion of abnormal GEFV than patients with evidence against GERD (43 [24.7%] *vs.* 4 [6.1%]; *P* < 0.05).

### Baseline characteristics of different GEFV grades and comparison of demographic findings between abnormal GEFV and normal GEFV groups

As shown in Table 2, the proportion of males increased gradually from GEFV I to GEFV IV (20 [21.3%], 50 [34.5%], 17 [42.5%] and 22 [53.7%], respectively) and it was significantly higher in the abnormal GEFV group than in the normal GEFV group (39 [48.1%] *vs.* 70 [29.3%], *P* = 0.002). The median age of the abnormal GEFV group was significantly higher than that of the normal GEFV group (63.0 [51.5–71.5] *vs.* 60.0 [51.0–66.0], *P* = 0.031). Also, age showed a positive correlation with GEFV grade (r = 0.178, *P* = 0.001). The mean BMI was similar between the normal GEFV and abnormal GEFV groups, and it showed no correlation with GEFV grade (r = 0.097, *P* = 0.084).

**Table 2:** Demographic and 24-h MII-pH Data and HRM Parameters of 320 Patients with GERD Symptoms according to GEFV Grade.

	GEFV I (n = 94)	GEFV II (n = 145)	GEFV III (n = 40)	GEFV IV (n = 41)	*P*-value	Normal GEFV (n = 239)	Abnormal GEFV (n = 81)	*P*-value
Age	56.0(51.0–63.0)	61.0(52.0–67.0)	59.0(48.5–65.0)	67.0(54.5–77.5)	0.001	60.0(51.0–66.0)	63.0(51.5–71.5)	0.031
Male gender, n(%)	20(21.3%)	50(34.5%)	17(42.5%)	22(53.7%)	0.002	70 (29.3%)	39(48.1%)	0.002
BMI	23.0 ± 3.8	24.0 ± 3.7	23.3 ± 3.9	24.5 ± 3.5	0.092	23.6 ± 3.7	23.9 ± 3.8	0.556
**24 h pH-MII parameters**								
AET%	0.9(0.2–2.9)	1.7(0.6–4.8)	1.5(0.3–4.2)	6.8(3.7–10.3)	< 0.001	1.3(0.3–4.2)	4.2(1.5–7.4)	< 0.001
Upright AET (%)	0.9(0.2–3.1)	2.5(0.6–6.1)	1.8(0.5–5.6)	7.3(3.9–11.1)	< 0.001	1.5(0.4–5.0)	4.9(1.3–9.4)	< 0.001
Recumbent AET (%)	0.1(0.0–0.6)	0.3(0.0–1.4)	0.5(0.0–1.4)	4.9(1.6–10.9)	< 0.001	0.2(0–1.4)	1.6(0.1–5.7)	< 0.001
MNBI(Ω)	2205.1(1944.4–2739.0)	2270.6(1777.1–2647.9)	2147.3(1662.6–2588.8)	1922.8(1514.7–2329.7)	0.015	2228.5(1794.8–2705.3)	2068.3(1658.4–2432.4)	0.012
PSPWI (%)	42.1(27.8–48.3)	29.9(23.3–38.4)	21.8(16.2–31.1)	18.2(11.7–28.2)	< 0.001	33.3(25.0–44.0)	19.7(13.9–29.0)	< 0.001
Bolus exposure (%)	0.5(0.3–0.8)	0.5(0.2–0.9)	0.6(0.3–1.2)	0.5(0.2–0.8)	0.608	0.5(0.2–0.9)	0.5(0.3–1.0)	0.635
Reflux event (n)	33.5(19.5–70.0)	36.0(17.5–59.0)	45.0(22.3–77.5)	40.0(21.5–69.5)	0.361	35.0(18.0–65.8)	43.5(22.0–76.0)	0.133
**HRM parameters**								
EGJ tone								
EGJ-CI(mmHg.cm)	14.4(9.6–28.4)	16.8(11.4–25.4)	17.0(8.6–28.7)	17.9(12.1–24.4)	0.903	16.4(10.3–26.1)	17.7(10.6–24.5)	0.921
EGJP-insp(mmHg)	42.9(27.9–59.7)	42.3(28.7–58.7)	39.3(23.3–58.2)	38.7(24.7–52.3)	0.548	42.3(28.3–59.3)	39.0(24.2–55.8)	0.155
EGJP-exp(mmHg)	45.8(31.5–66.3)	41.3(25.0–59.8)	39.3(21.4–60.8)	45.0(27.7–55.8)	0.486	43.0(26.0–62.7)	41.3(24.0–58.0)	0.377
**EGJ morphology**					0.001			0.011
EGJ I	31(33.0%)	36(24.8%)	7(17.5%)	3(7.3%)		67(28.0%)	10(12.3%)	
EGJ II	52(55.3%)	89(61.4%)	29(72.5%)	28(68.3%)		141(59.0%)	57(70.4%)	
EGJ III	11(11.7%)	20(13.8%)	4(10.0%)	10(24.4%)		31(13.0%)	14(17.3%)	
**Esophageal body motility 0.077 0.216**								
Normal	40(42.6%)	59(40.7%)	17(42.5%)	12(29.3%)		99(41.4%)	29(35.8%)	
Fragmented peristalsis	16(17.0%)	19(13.1%)	6(15.0%)	3(7.3%)		35(14.6%)	9(11.1%)	
IEM	38(40.4%)	67(46.2%)	17(42.5%)	26(63.4%)		105(43.9%)	43(53.1%)	
**Endoscopy**					< 0.001			< 0.001
Normal	77(81.9%)	30(20.7%)	12(30.0%)	3(7.3%)		107(44.8%)	15(18.5%)	
LA-A&B	16(17.0%)	102(70.3%)	26(65.0%)	33(80.5)		118(49.4%)	59(72.8%)	
LA-C&D&BE	1(1.1%)	13(9.0%)	2(5.0%)	5(12.2%)		14(5.9%)	7(8.6%)	

Values are presented as the median and interquartile range, except for BMI (mean and standard deviation), male gender, EGJ morphology, Esophageal body motility, and Endoscopy (n and %).

*MII-pH* multichannel intraluminal impedance-pH, *HRM* high-resolution manometry, *GERD* gastroesophageal reflux disease, *GEFV* gastroesophageal flap valve, *BMI* body mass index, *BE* Barrett’s esophagus, *AET* acid exposure time, *MNBI* mean nocturnal baseline impedance, *PSPWI* post-reflux swallow-induced peristaltic wave index, *EGJ* esophagogastric junction, *EGJ-CI* EGJ-contractile integral, *EGJP-insp* inspiratory EGJ pressure, *EGJP-exp* expiratory EGJ pressure, *IEM* ineffective esophageal motility.

*P* < 0.05 was considered to be significant.

### Comparison of MII-pH parameters between abnormal GEFV and normal GEFV groups

As shown in Table 2, the abnormal GEFV group had a significantly higher total AET%, upright AET%, and recumbent AET% than the normal GEFV group (4.2 [1.5–7.4] *vs.* 1.3 [0.3–4.2], 4.9 [1.3–9.4] *vs.* 1.5 [0.4–5.0], and 1.6 [0.1–5.7] *vs*. 0.2 [0–1.4], respectively; all *P* < 0.001). Moreover, MNBI and PSPWI were significantly lower in the abnormal GEFV group than in the normal GEFV group (2068.3 [1658.4–2432.4] Ω *vs.* 2228.5 [1794.8–2705.3] Ω, *P* = 0.012; 19.7 [13.9–29.0] % *vs.* 33.3 [25.0–44.0] %, *P* < 0.001, respectively). Reflux events and bolus exposures were similar between the abnormal GEFV and normal GEFV groups.

### Comparison of HRM findings between abnormal GEFV and normal GEFV groups

The proportion of Type I EGJ morphology gradually decreased from GEFV I to GEFV IV (31 [33.0%] *vs*. 36 [24.8%] *vs.* 7 [17.5%] *vs.* 3 [7.3%], respectively), and it was significantly higher in the normal GEFV group than in the abnormal GEFV group (67 [28.0%] *vs.* 10 [12.3%], *P* = 0.011). However, the values for EGJ-CI, EGJP-insp, EGJP-exp, and the proportion of normal esophageal motility were similar between the abnormal GEFV and normal GEFV groups.

### Correlation of GEFV grade with MII-pH and HRM findings

**Figure 2:**
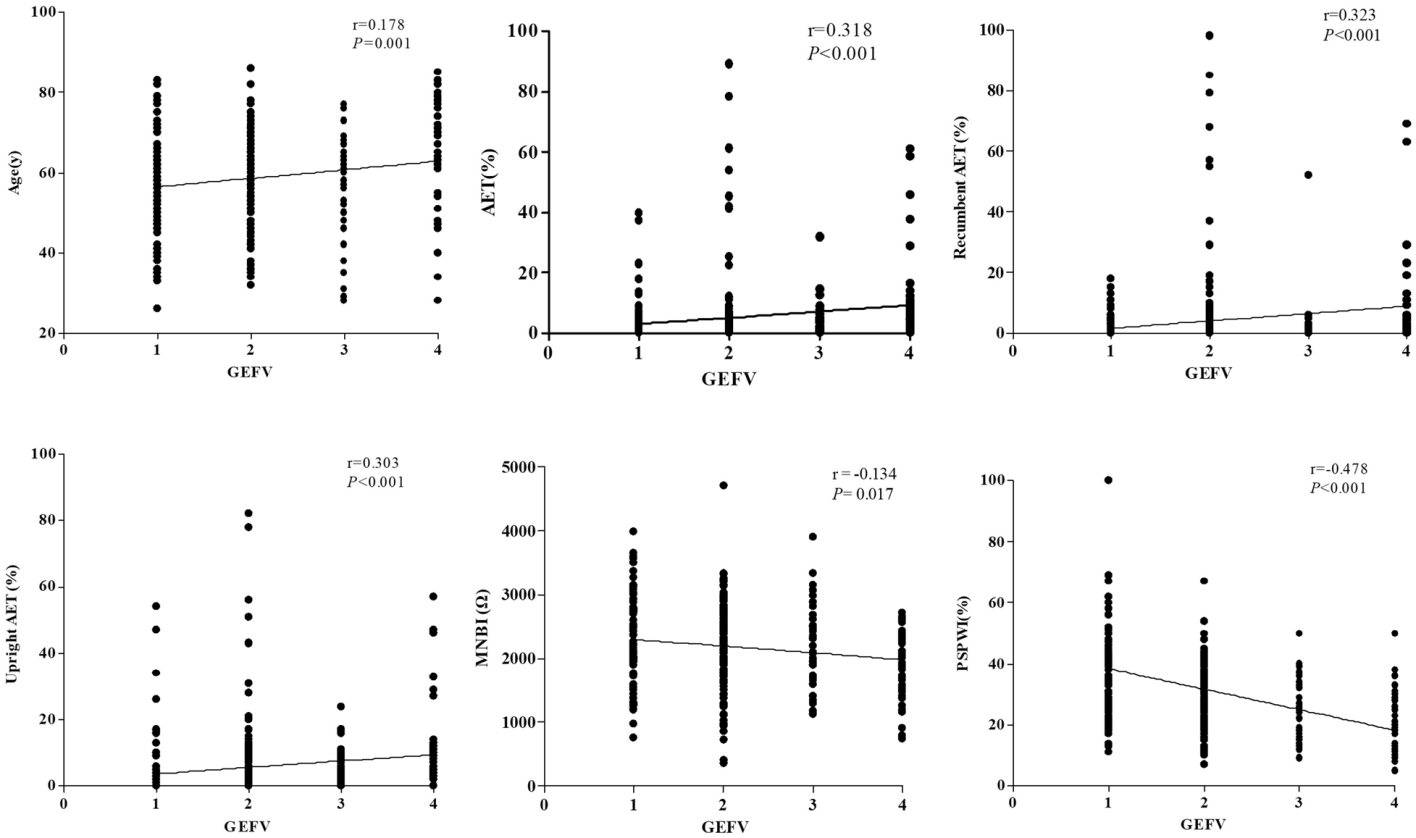
Associations of GEFV grade with age, total AET%, upright AET (%), recumbent AET (%), MNBI, and PSPWI. *AET* acid exposure time, *GEFV* gastroesophageal flap valve, *MNBI* mean nocturnal baseline impedance, *PSPWI* post-reflux swallow-induced peristaltic wave index.

**Figure 3:**
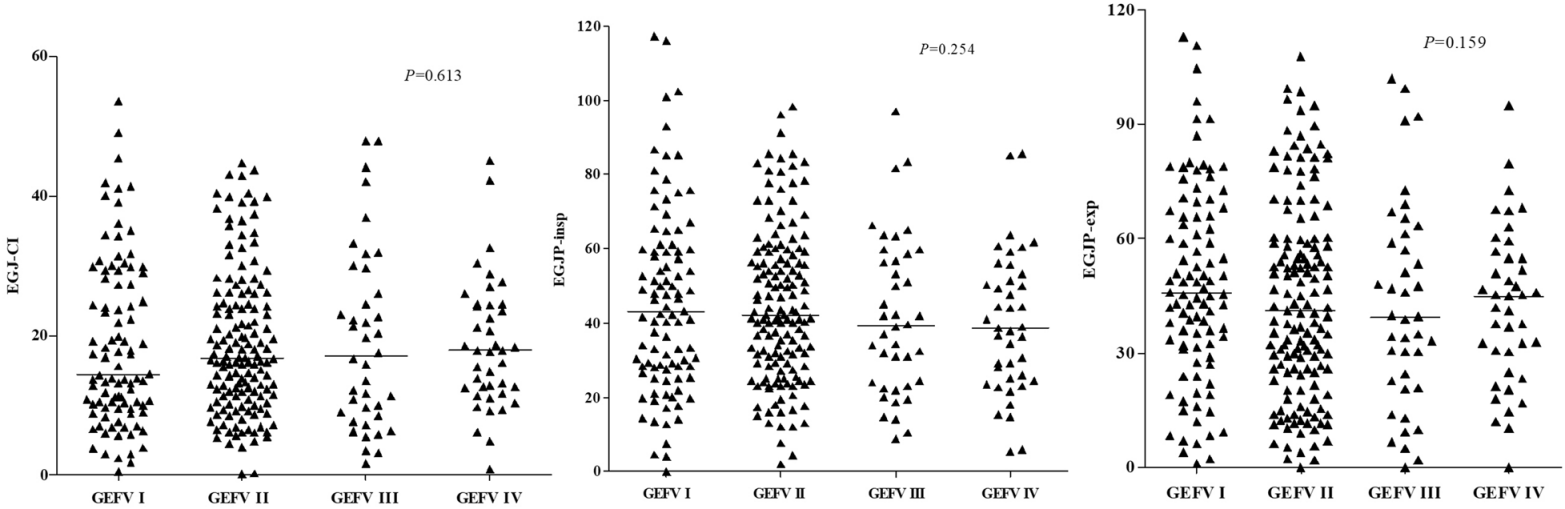
Associations of GEFV grade with EGJ-CI, EGJP-insp, and EGJP-exp. *GEFV* gastroesophageal flap valve, *EGJ* esophagogastric junction, *EGJ-CI* EGJ-contractile, *EGJP-insp* inspiratory EGJ pressure, *EGJP-exp* expiratory EGJ pressure.

GEFV grade was positively correlated with total AET%, recumbent AET%, and upright AET% (r = 0.318, r = 0.323, and r = 0.303, respectively; all *P* < 0.001). PSPWI had a negative correlation with GEFV grade (r = − 0.478, p < 0.001). GEFV grade had a weak correlation with MNBI and EGJ morphology (r = − 0.134, *P* = 0.017; r = 0.172, *P* = 0.002; respectively) (Fig. 2). There were no significant differences in EGJ-CI, EGJP-insp and EGJP-exp among the different GEFV grades (Fig. 3).

### Diagnostic value of abnormal GEFV for conclusive evidence of GERD

Patients with an abnormal GEFV had a significantly greater risk of conclusive evidence of GERD compared to patients with a normal GEFV (OR 3.035, 95% CI 1.758–5.240, *P* < 0.001) in patients with GERD symptoms. Further, when identifying patients with conclusive evidence of GERD in patients with GERD symptoms, abnormal GEFV had an accuracy of 70.9% (95% CI 66.0–75.8%), sensitivity of 42.5% (95% CI 31.7–53.3%), specificity of 80.4% (95% CI 75.3–85.5%), positive predictive value of 42.0% (95% CI 31.2–52.8%), and negative predictive value of 80.8% (95% CI 75.9–85.7%), respectively.

## Discussion

Because of the heterogeneity, there is no simple definition and classification of GERD. Instead, there are many proposed diagnostic classifications for GERD. In one of the most accepted classifications, patients with GERD symptoms are categorized as having an EE or non-erosive reflux disease (NERD)^15^. The Lyon Consensus was published to classify GERD based on endoscopy, biopsy, ambulatory reflux monitoring, and HRM findings. Moreover, novel parameters such as MNBI and PSPWI were also included^2^. However, GEFV grading has not yet been included in the Lyon Consensus. Also, there has been no study on the relationship between GEFV grade and the Lyon Consensus classification.

In the present study, we evaluated the association between GEFV grade and patients with GERD symptoms as classified by the Lyon Consensus. An abnormal GEFV was present in 25.3% of all patients with GERD symptoms in our study, which was in line with previous studies showing that the proportion of abnormal GEFV in patients with GERD symptoms ranged from 25.0 to 36.2%^7,16–18^. And the proportion of abnormal GEFV was 42.5% in patients with conclusive evidence of GERD, consistent with previous papers reporting that the percentage of abnormal GEFV in GERD patients was from 34.9 to 52.6%^7,16^. Further, we demonstrated that an abnormal GEFV was a significant risk factor for conclusive evidence of GERD in patients with GERD symptoms. The specificity of abnormal GEFV for conclusive evidence of GERD was good (80.4%). And a recent meta-analysis showed that the pooled specificity of an abnormal GEFV for the diagnosis of EE in patients with symptomatic GERD was 75.7%^19^. However, the present study and the previous meta anylysis^19^ showed that the sensitivity of abnormal GEFV for GERD was low, resulting from the heterogeneity of GERD pathophysiology, which included not only EGJ barrier function but also mucosal integrity, esophageal peristalsis, etc. Our study firstly used abnormal GEFV to detect conclusive evidence of GERD in patients with GERD symptoms as classified by the Lyon Consensus, which proposed stricter criteria for GERD diagnosis to avoid inappropriate use of PPIs.

The Lyon Consensus not only proposed stricter criteria for GERD diagnosis but also included novel metrics on MII-pH and HRM^2^. Therefore, we further explored the association between GEFV grading and these traditional and novel parameters. In the present study, AET% showed a significant positive correlation with GEFV grade, which was consistent with the findings of previous studies^7,17^. Kim et al. found that the incidence of distal gastroesophageal reflux was significantly higher in subjects with an abnormal GEFV^17^. Xie et al. also found that the AET% was lower in patients with GEFV grade I^7^. In addition, previous studies have shown that GEFV III/IV grades were associated with a higher prevalence of EE and BE^17,18,20^. The reason could be that the GEFV is a part of the anti-reflux barrier and higher grades of GEFV indicate anatomical disturbance in the EGJ, which increases the risk of reflux^7^.

The MNBI and the PSPWI were regarded as supportive evidence for the diagnosis of GERD in the Lyon Consensus^2^. The MNBI reflects the esophageal mucosal integrity, the link to reflux symptoms, alterations in tight junctions, and intercellular space^21,22^. The PSPW suggests whether primary peristalsis is initiated in the esophagus on stimulation by reflux and evaluates the esophageal chemical clearance^14,23^. These two parameters have been found to increase the diagnostic yield of MII/pH for GERD, even if the patients are on PPI therapy^24–26^. In addition, abnormal PSPWI and MNBI have been shown to be associated with a satisfactory response to medical treatment for GERD^27^. Ribolsi et al. showed that pathological AET, absent contractility on HRM, and the presence of type 2 or 3 EGJ significantly correlated with pathological MNBI values^3^. To the best of our knowledge, there has been no study on the relationship between GEFV grade and MNBI/PSPWI. The current study showed that MNBI and PSPWI were significantly lower in the abnormal GEFV group, and these parameters had significant negative correlations with GEFV grades (from I to IV). Therefore, we believe that abnormal GEFV reflects a poor anti-reflux barrier, which leads to a higher reflux burden and results in a lower MNBI and PSPWI. These results indicate that the GEFV and its grade can serve as supportive evidence for the diagnosis of GERD.

The EGJ plays a crucial role as an anti-reflux barrier. EGJ morphology, which is determined by the spatial separation between CD and LES, is an important determinant of the EGJ barrier function^28^. EGJ-CI is a novel parameter reflecting a contraction of the EGJ at rest and has shown to be decreased in GERD patients compared with those with functional heartburn^11^. Xie et al. suggested that the GEFV grade could reflect EGJ morphology on HRM and that EGJ type III correlated with GEFV grade IV, but the GEFV grades had no impact on the EGJ-CI, EGJP-exp, and EGJP-insp. The authors believed that the reason might be that the GEFV was not the only structure contributing to EGJ contractility^7^. Our study also showed that the type I EGJ morphology was more common in patients with a normal GEFV than in those with an abnormal GEFV. However, the values for EGJ-CI, EGJP-insp, and EGJP-exp were similar between the abnormal GEFV and normal GEFV groups. In addition, GEFV grade had no impact on esophageal body motility.

In the current study, the abnormal GEFV group had a significantly higher proportion of males, consistent with a previous study showing that the male gender was a significant risk factor for abnormal GEFV^29^. Among patients with GERD symptoms, those in the abnormal GEFV group were significantly older, which was in line with a previous study showing that among patients with laryngopharyngeal reflux, those with an abnormal GEFV were significantly older than those with a normal GEFV^30^.

The present study showed that inter-observer agreement of GEFV grading was 92.1%, which was consistent with previous studies demonstrating that the rate of agreement of GEFV grading ranged from 80.0 to 92.4%^30–32^. And disagreement was never more than one grade and usually occurred in the classification between GEFV I and II or between GEFV III and IV^31^. In addition, to our knowledge, there has been no study to evaluate if sedation has effects on GEFV abnormality. In the present study, the concordance rate of GEFV grading was excellent between intravenous anesthesia and topical anesthesia in 24 patients, who had undergone twice EGD examination under intravenous anesthesia and topical anesthesia respectively within 2 years. In addition, the present study showed that sedation was not a risk factor for abnormal GEFV. These findings indicated that sedation might not affect GEFV abnormality. However, further prospective and multi-center studies are needed on this issue.

Of note, due to the heterogeneity of GERD pathophysiology, GEFV grading could not replace but aid MII-pH monitoring, which is considered to be the gold standard for the identification of reflux events^2^. However, 24 h MII-pH has not been widely available in the clinic due to time consuming, inconvenience and discomfort, and the cost burden^2^. Our study showed that abnormal GEFV was a significant risk factor for conclusive evidence of GERD. In addition, GEFV grading as a simple and reproducible classification during routine endoscopy was associated with reflux burden and could be a good reflection of EGJ morphology. Moreover, to our knowledge, the present study firstly demonstrated that GEFV grading was correlated with MNBI and PSPWI, which were two novel impedance-detected parameters included in the Lyon Consensus, reflecting mucosal integrity and esophageal peristalsis respectively^2^. Therefore, GEFV grading might be referred to as supportive evidence of GERD in patients with typical GERD symptoms.

This study has several strengths. First, the number of patients was relatively large, with over 300 patients being evaluated. Second, all patients received endoscopy, 24-h MII-pH monitoring, and HRM. Third, this study was the first to show the association between GEFV grade and the classification of GERD by Lyon Consensus, and the association of GEFV with the MNBI and PSPWI, two novel metrics on MII-pH. However, this study has some limitations. It was a single-center retrospective study, which may be associated with selection bias. In addition, some patients underwent endoscopy after PPI therapy. Thus, there is a possibility of misclassification of the esophagitis on endoscopy.

In summary, an abnormal GEFV was a significant risk factor for conclusive evidence of GERD in patients with GERD symptoms, and patients with an abnormal GEFV had a higher reflux burden, lower MNBI, and lower PSPWI. We recommend that GEFV grading should be determined during endoscopy and can be regarded as supportive evidence for the diagnosis of GERD.

## Data Availability

The datasets analyzed during the current study are available from the corresponding authors on reasonable request.
